# Parents’ psychosocial needs during the child’s hospitalization in pediatric intensive care units (PICU): a systematic review

**DOI:** 10.1192/j.eurpsy.2024.1402

**Published:** 2024-08-27

**Authors:** I. Kalogerogianni, A. Zartaloudi, E. Dousis, E. Evangelou, C. Dafogianni, E. Vlachou, M. Polikandrioti, I. Koutelekos

**Affiliations:** ^1^ University of West Attica; ^2^Greek Health System, Athens, Greece

## Abstract

**Introduction:**

The admission and hospitalization of a child in a Pediatric Intensive Care Unit (PICU) creates stress and anxiety in the family. The family is called upon to make important decisions about the child’s treatment, while roles within the family environment are disrupted.

**Objectives:**

The investigation of the psychosocial needs of the relatives of hospitalized children in the NICU.

**Methods:**

We conducted a systematic review of studies published until the end of 2022 in the Greek and English languages in the databases “Pubmed”, “Scopus” and “Iatrotec” with the following keywords: “Pediatric Intensive Care Unit”, “Socio-psychological Needs’ and ‘Parents’.

**Results:**

Of the 26 studies found, 5 studies met the inclusion-exclusion criteria and were included in the review. The most frequently mentioned psychosocial needs of the parents were: (1) the need for complete, immediate and honest information regarding the health status of their hospitalized child and the changes in their condition, (2) the need to provide comfort to the parents during duration of their child’s hospitalization, (3) the parents’ need for psychological support and guidance regarding the care of their hospitalized child, (4) the feeling of security regarding the care provided, and (5) the need for frequent contact with the hospitalized child. Also, it was observed that the medical and nursing staff underestimated some needs of the parents, such as the need for closeness, while there were others that we underestimated, such as the religious needs.

**Conclusions:**

Parents present increased psychosocial needs during their child’s hospitalization in the PICU. Nursing staff play an important role in supporting relatives by providing family-centered care.

**Disclosure of Interest:**

None Declared

